# Improving Solubility and Permeability of Hesperidin through Electrospun Orange-Peel-Extract-Loaded Nanofibers

**DOI:** 10.3390/ijms24097963

**Published:** 2023-04-27

**Authors:** Magdalena Paczkowska-Walendowska, Andrzej Miklaszewski, Judyta Cielecka-Piontek

**Affiliations:** 1 Department of Pharmacognosy, Poznan University of Medical Sciences, Rokietnicka 3, 60-806 Poznan, Poland; jpiontek@ump.edu.pl; 2 Faculty of Mechanical Engineering and Management, Institute of Materials Science and Engineering, Poznan University of Technology, 60-965 Poznan, Poland; andrzej.miklaszewski@put.poznan.pl

**Keywords:** orange peel extract, nanofibers, hesperidin, solubility, permeability

## Abstract

Orange peel, which is a rich source of polyphenolic compounds, including hesperidin, is produced as waste in production. Therefore, optimization of the extraction of hesperidin was performed to obtain its highest content. The influence of process parameters such as the kind of extraction mixture, its temperature and the number of repetitions of the cycles on hesperidin content, the total content of phenolic compounds and antioxidant (DPPH scavenging assay) as well as anti-inflammation activities (inhibition of hyaluronidase activity) was checked. Methanol and temperature were key parameters determining the efficiency of extraction in terms of the possibility of extracting compounds with the highest biological activity. The optimal parameters of the orange peel extraction process were 70% of methanol in the extraction mixture, a temperature of 70 °C and 4 cycles per 20 min. The second part of the work focuses on developing electrospinning technology to synthesize nanofibers of polyvinylpyrrolidone (PVP) and hydroxypropyl-β-cyclodextrin (HPβCD) loaded with hesperidin-rich orange peel extract. This is a response to the circumvention of restrictions in the use of hesperidin due to its poor bioavailability resulting from low solubility and permeability. Dissolution studies showed improved hesperidin solubility (over eight-fold), while the PAMPA-GIT assay confirmed significantly better transmucosal penetration (over nine-fold). A DPPH scavenging assay of antioxidant activity as well as inhibition of hyaluronidase to express anti-inflammation activity was established for hesperidin in prepared electrospun nanofibers, especially those based on HPβCD and PVP. Thus, hesperidin-rich orange peel nanofibers may have potential buccal applications to induce improved systemic effects with pro-health biological activity.

## 1. Introduction

*Citrus aurantium* (L.) (orange) is the world’s most widely grown and traded citrus species. The orange fruit is usually eaten whole or made into juice after peeling the outer peel. This peeling process leads to the generation of considerable peel waste [1]. Due to the content of bioactive compounds ((1). essential oil with limonene; (2). polyphenols as flavan-3-ols: catechin and epicatechin; flavanols: quercetin, rutin and kaempferol; phenolic acids: caffeic acid; flavanones: hesperetin, hesperidin, naringenin and naringin; and flavone: luteolin; and (3). phenylethylamine alkaloids including p-synephrine), the food and pharmaceutical industries use orange peels to prepare extracts [2,3]. Orange peel extracts have been used to make cosmetics, aromas and taste enhancers for food and drinks [4]. Finally, many health-promoting properties of using orange peel preparations have been noticed. It has been shown that there is enough hesperidin in the plant material, as the glycosidic form is dominant compared to aglycone hesperetin, such that the orange peel can be used to isolate pure hesperidin [5,6].

Hesperidin has been recently extensively evaluated for its health-promoting effects to reveal its antioxidant, anti-inflammatory, anticancer, antiviral, cardio-protective and neuroprotective properties [7]. Despite hesperidin’s broad pharmacological activity, the low water solubility limits its bioavailability [8]. After oral intake, hesperidin is hydrolyzed by the gut microbial in the small intestine and mainly in the colon into the aglycone form (hesperetin) and then converted to glucuronides in the large intestine. About 3 h after consumption, hesperetin is present in plasma as glucuronides (87%) and sulphaglucuronides (13%) [7,9]. Overall, the bioavailability of hesperidin is estimated at 20% [10]. In turn, low bioavailability may limit its use.

In this nano era, numerous top-down and bottom-up nano methods have been introduced into the applications of manipulating drugs’ encapsulation, dissolution, permeation and controlled release behaviors [11]. Some approaches, such as the encapsulation and micronization of hesperidin, have been proposed, particularly for drug production, to improve its bioavailability, stability and controlled release [12,13,14]. In addition, a challenge was undertaken to increase the oral bioavailability of hesperidin through its amorphization [15,16]. Among nano-techniques, electrospinning is drawing increasing attention due to its powerful capability of preparing nanocomposites [17] and complicated nanostructures [18] and the unique properties of the resultant nanofibers [19]. Electrospun nanofiber-based drug delivery systems have shown tremendous progress over classical systems due to the large contact surface area with body fluids, adjustable porosity, mechanical strength, biocompatibility, high drug loading and customizable release properties [20]. Drugs as well as other functional molecules are added directly to working fluids with flame-forming polymer matrices, so the selection of polymers is crucial and can affect the final properties of the nanofibers. One such additive may be cyclodextrin, which affects not the quality of the fibers themselves but their functionality, increasing the solubility of the encapsulated active compounds [21]. There are reports of the production of hesperidin-loaded polyacrylonitrile/polyethylene oxide nanofibers and hesperidin-loaded lipid polymer hybrid nanoparticles, which were intended for topical use [22,23].

Thus, in this work, an attempt was made to produce rapidly soluble HPβCD/PVP-based nanofibers containing hesperidin-rich orange peel extract to improve its solubility and permeability. Full characterization of physicochemical properties as well as an evaluation of pharmaceutical properties has been carried out. The optimization of the orange peel extraction process preceded these studies.

## 2. Results and Discussion

### 2.1. Optimization of the Orange Peel Extraction Process and Characterization of Biological Activity

In recent years, interest in using herbal medicines in treating and preventing various diseases has increased worldwide, although in most cases, their quality may be questionable. Hence, there is a need to standardize plant product requirements and look for reliable tools to verify their quality. Therefore, there is an increasing emphasis on understanding the extraction process and determining the critical parameters of the process. In this work, a full factorial design model was created to assess the effectiveness of the extraction process. The influence of methanol content in the extraction mixture, its temperature and the number of repetitions of the cycle on hesperidin and total polyphenol content and the antioxidant and anti-inflammatory activity of extracts was assessed.

**Figure 1:**
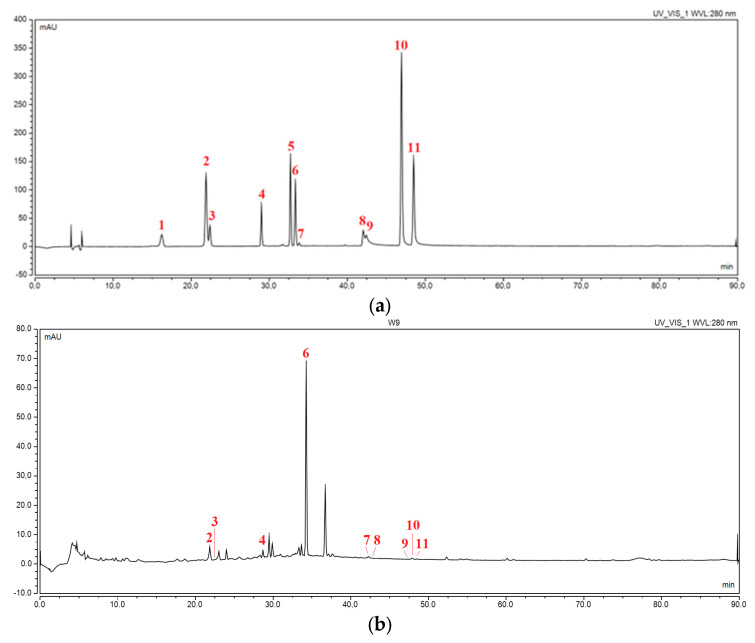
Chromatogram of (**a**) a mixture of standards at a wavelength of 280 nm (1—catechin, 2—caffeic acid, 3—epicatechin, 4—rutin, 5—naringin, 6—hesperidin, 7—luteolin, 8—quercetin, 9—naringenin, 10—kaempferol, 11—hesperetin); (**b**) extract E9 at a wavelength of 280 nm.

The separation and the determination of 11 active compounds (flavan-3-ols: catechin and epicatechin; flavanols: quercetin, rutin and kaempferol; phenolic acids: caffeic acid; flavanones: hesperetin, hesperidin, naringenin and naringin; and flavone: luteline) contained in the orange peel extracts, as well as all concentration changes in hesperidin during dissolution and permeability studies of electrospun nanofibers, were carried out using high-performance liquid chromatography supported by a photodiode array detector. The retention times of all 11 compounds were compared with the retention times and UV spectra of the reference substances (Figure 1a,b). The HPLC method was validated according to ICH guidelines, and validation parameters are collected in Table S1 (Supplementary Materials). Therefore, the developed method may be a reference for determining the concentrations of the polyphenols mentioned above in plant material and pharmaceutical dosage forms containing orange peel.

Using the linearity equation of all 11 reference substances, it was possible to determine the content of all active compounds in all 9 extracts (Table S2, Supplementary Materials), whereas up to the DoE model, only the hesperidin content was determined (Table 1). Based on the Pareto diagram (Figure S1, Supplementary Materials), it can be indicated that the percentage of methanol in the extraction mixture and the extraction temperature are statistically significant factors affecting the hesperidin content. Moreover, both effects have a positive sign, i.e., with the increase in the percentage of methanol in the extraction mixture and with the rise in temperature, the hesperidin content increases. In addition to the hesperidin content, all extracts’ total polyphenol content (TPC) was determined (Table 1). On the other hand, analyzing the Pareto diagram (Figure S2, Supplementary Materials), only the percentage of methanol is a statistically significant factor affecting the total phenolic content. This effect also exhibits a positive sign.

It is critical to ascertain the biological activity of the produced extracts. For this, the antioxidant (measured by four methods: DPPH, ABTS, CUPRAC and FRAP) potential, as well as the anti-inflammatory activities (measured as inhibition of the hyaluronidase enzyme), was evaluated. The complete results are shown in Table 2. Analyzing the Pareto diagram (Figures S3 and S4, Supplementary Materials), it was noticed that the percentage of methanol in the extraction mixture and the temperature process are statistically significant factors affecting both antioxidant (measured by the DPPH method) and anti-inflammatory activities. Interestingly, the effects had different signs, i.e., with increasing methanol concentration and temperature, antioxidant activity increased (expressed as a decrease in IC_50_), while anti-inflammatory activity decreased (observed as an increase in IC_50_).

**Figure 2:**
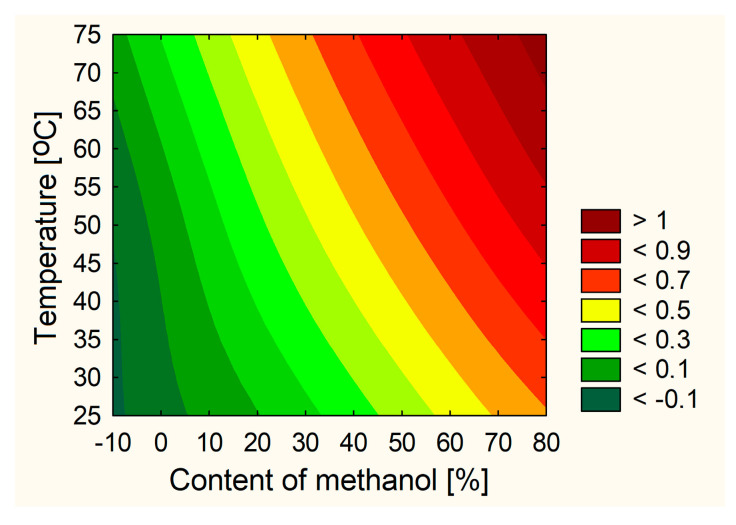
Prediction of DoE model of optimization of the extraction process.

The technical aspects of the extraction procedure that produced the extract with the best characteristics and the highest activity could be identified based on the test results and statistical analyses. None of the analyzed cases showed that the number of extraction cycles was a critical parameter. Therefore, based on the utility contour profiles model, including all measured outputs, it was possible to predict the model and indicate optimized parameters of the orange peel extraction process, which are 70% of methanol in the extraction mixture, a temperature of 70 °C and 4 cycles per 20 min (statistically insignificant parameter) (Figure 2). It was decided to leave the middle number of cycles due to, on the one hand, the previously demonstrated dependence on the correlation between the duration of the process and the extraction efficiency [4] and, on the other hand, it not having the highest level of impact in reducing the cost of the process.

The E10 extract was created using optimized process parameters, and its activity was assessed to validate the model. The second phase of the experiment, which involved the creation of electrospun nanofibers, utilized the E10 extract.

### 2.2. Optimization of the Electrospinning Process of Nanofibers Involving Orange Peel Extract

#### 2.2.1. Preparation and Identification of Electrospun Nanofibers

In the second part of the work, an attempt was made to optimize the electrospinning process. In a previous study, a significant effect of PVP and HPβCD on the physicochemical properties of nanofibers was demonstrated; therefore, the impact of these two excipients on the quality of nanofibers rich in orange peel extract was also examined in this study. Moreover, it was noticed that the addition of dichloromethane to methanol improves the quality of nanofibers [24]; hence, the content of dichloromethane in the mixture was also tested. The surface tension and viscoelastic properties of the polymer solution are among the key parameters of the electrospinning process. Therefore, before the electrospinning process, the rheological behavior of the prepared solutions expressed by their viscosity was checked (Figure S5, Supplementary Materials). As expected, the viscosity increased with increasing PVP concentration (statistically significant relationship resulting from the Pareto chart—Figure S6, Supplementary Materials) and HPβCD concentration (statistically insignificant relationship), given that both substances are known to have viscosity-raising properties [25,26]. At the same time, the concentration of dichloromethane did not affect the viscosity of the mixtures. Most solutions showed viscosities in the conventional range of ~5 Pa·s [27]. Then, the electrospinning process with the same conditions regarding the applied voltage, flow rate and distance between the collector and the needle tip was examined, leading to the production of a continuous length of fibers.

The produced electrospun nanofibers were examined using SEM to determine their morphology, XRPD to show structural characteristics and ATR-IR to identify any potential intermolecular chemical bond formation.

**Figure 3:**
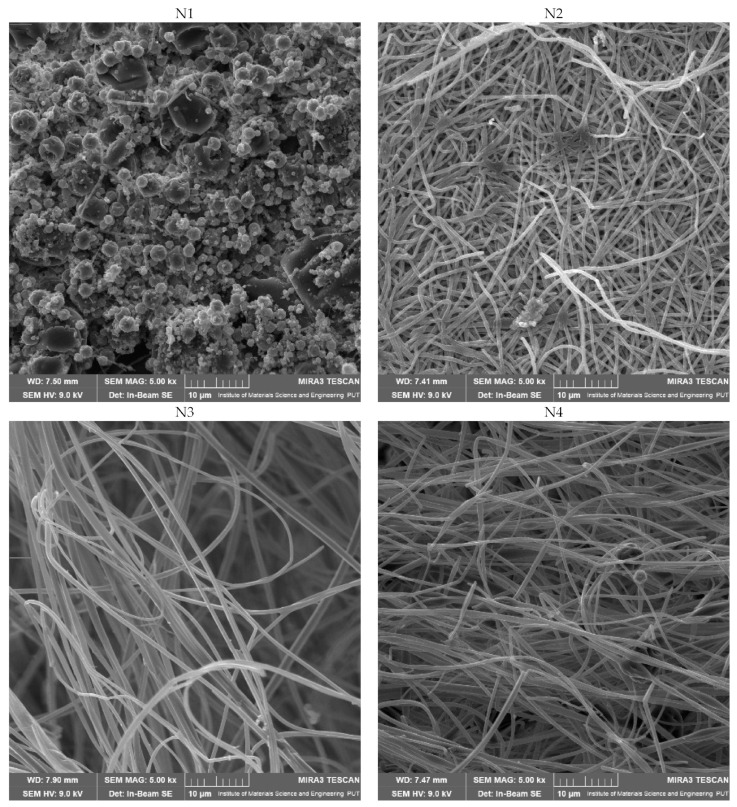

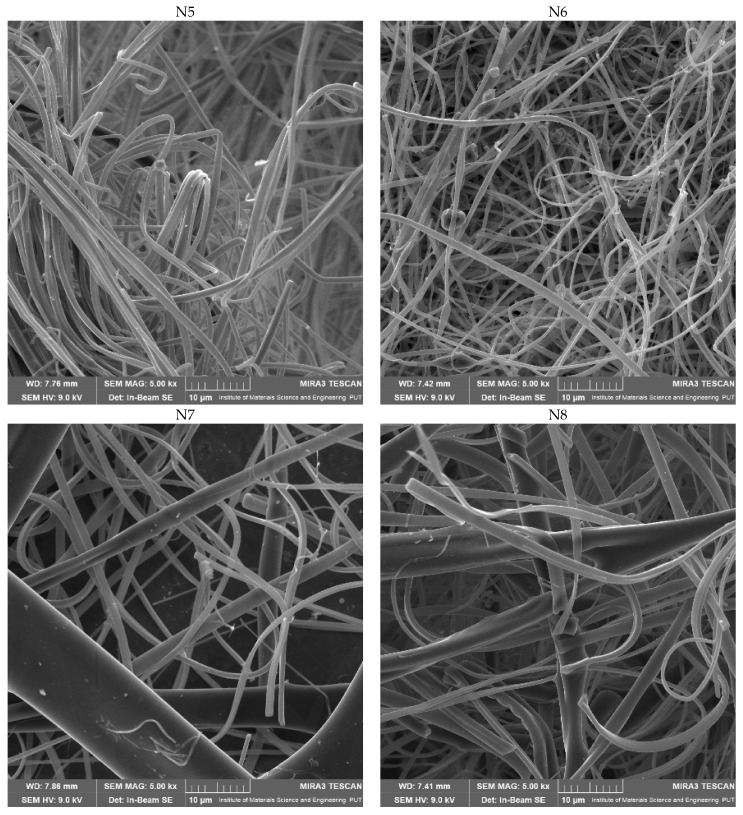

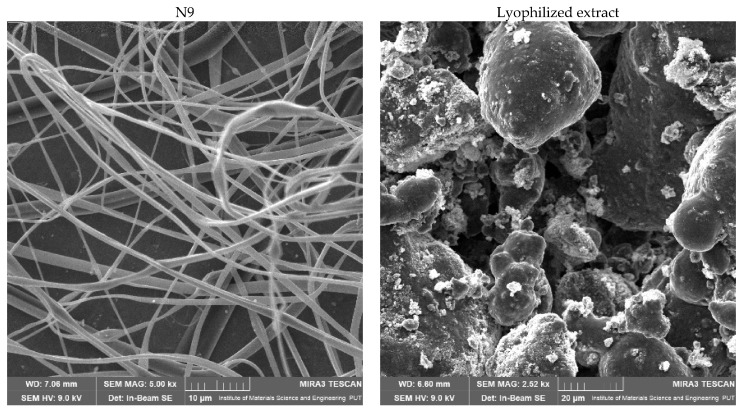
SEM images of nanofibers 1–9 and lyophilized extract.

Figure 3 shows that the production of systems 2–9 went well and that good-looking nanofibers were obtained. In contrast, in the first system, round particles/beads, not resembling fibers, were obtained. Furthermore, in the case of systems no. 1, 4 and 7, where the concentration of PVP was the lowest, some beads could be observed. Due to the low viscosity of the polymer solution, which caused the development of beads at low concentrations instead of nanofibers, the surface tension was dominant. The more viscous polymer solution created continuous, homogeneous nanofibers when the concentration was increased [28]. Based on the Pareto chart, it was noticed that increasing the concentration of PVP in the mixture causes a decrease in the diameter of nanofibers, while HPβCD increases the size of the fibers; however, these effects were not statistically significant (Table 3; Figure S7, Supplementary Materials).

A critical relationship was observed between the viscosity of electrospinning solutions and the diameter of the nanofibers. A too-low concentration of polymers (in the case of system no. 1) resulted in the formation of beads instead of nanofibers (Figure 3), which was observed previously [29]. On the other hand, the diameter of the produced nanofibers increases with the increase in solution viscosity. When the solution viscosity is increased, the polymer beads are larger, the average distance between them is longer, and the diameter of the obtained fibers is increased [30,31]. Excessive increases in the diameter of the nanofibers should generally be avoided.

It is worth noting that the process ran smoothly in the case of N2–N5 nanofibers, which was also observed in the efficiency of the process (Table 4). A specific relationship was observed that the efficiency of the process was higher when obtaining fibers with a smaller diameter, with the highest efficiency of the process observed for N2–N5 nanofibers. Moreover, HPβCD statistically significantly affected the decrease in the efficiency of the electrospinning process (Figure S8, Supplementary Materials). In addition, it was shown that a certain increase in the dichloromethane content improves the efficiency of the process without affecting the size of the nanofibers.

**Figure 4:**
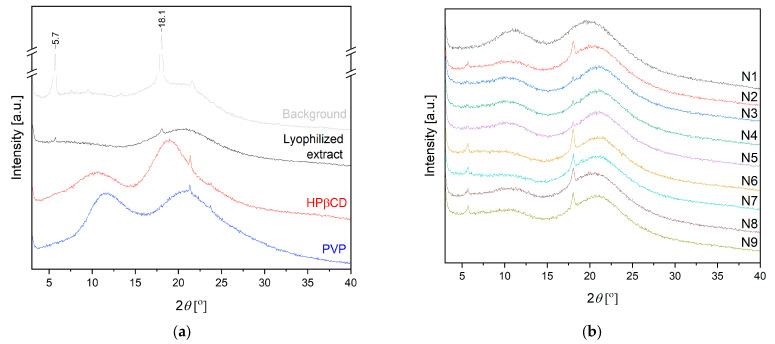
Diffractograms for lyophilized extract, HPβCD and PVP (**a**) and nanofibers N1–N9 (**b**).

Figure 4 presents the XRPD diffractogram of the lyophilized extract. The pattern for hesperidin is well described in the literature with sharp peaks characteristic of crystalline material at diffraction angles of 7.1, 12.2, 15.5, 18.3, 20.7, 21.3, 22.4 and 24.8° [16]. Based on the literature data, PVP, as well as HPβCD, exhibits broad peaks at around 13 and 20°, which refer to their amorphous nature [16,32]. Analyzing the pattern for the lyophilized extract, almost complete amorphization is visible, with only reflection at 18.1° corresponding to hesperidin. Based on previous experiments and literature data, the amorphous extract does not change its structure during electrospinning [33], which was also confirmed in the present studies (Figure 4b).

**Figure 5:**
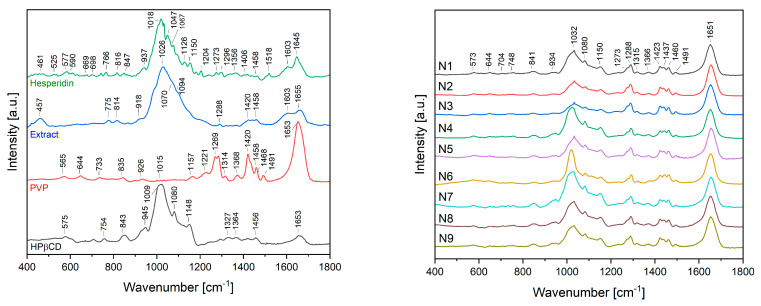
ATR-IR spectra for hesperidin, lyophilized extract, HPβCD, PVP and nanofibers N1–N9.

The ATR-IR spectra of nanofibers N1–N9 were analyzed and compared with the spectra of pure hesperidin, the lyophilized extract and excipients used for nanofiber fabrication, such as PVP and HPβCD (Figure 5).

The spectrum peak of carbonyl C=O stretch appeared at 1645 cm^−1^. The bands at 1518–1356 cm^−1^ are attributed to the aromatic C=C stretch and the aromatic C–O stretch at 1273, 1204, 1150, 1126 and 1067 cm^−1^. The ATR-IR spectra of native hesperidin showed characteristic bands because of the existence of different functional groups such as at 1645, 1518 and 1050 cm^−1^, which could be attributed to C=O stretching, C=C stretching and C–O stretching, respectively, as well as at 1018 cm^−1^ corresponding to C–C stretching in the rutinose ring and C–C stretching between glucose and rhamnose rings [16,34]. Most of the characteristic hesperidin bands are also observable in the spectrum of the extract. Finally, in the case of nanofiber fabrication materials, the spectrum of PVP had a peak at 1653 cm^−1^ proving the existence of stretching of C–O. The C-H bending and CH_2_ wagging were observed at 1420 cm^−1^ and 1269 cm^−1^, respectively. The peaks at 1015 and 565 cm^−1^ were identified as the CH_2_ rock and N–C=O bending, respectively [35]. Finally, the spectrum of HPβCD consisted of prominent absorption bands at 1653 cm^−1^ (H–OH bending vibrations), 1148 cm^−1^ (symmetric C–O–C stretching vibrations), 1080 cm^−1^ (C–O stretching vibrations), 1009 cm^−1^ (C–C stretching vibrations) and 947 and 847 cm^−1^ (presence of glucopyranose units). Furthermore, in the range of 1200–1500 cm^−1^, there are bands associated with bending vibrations of C–H and O–H bonds [36].

When analyzing the spectra of N1–N9 nanofibers, the most intense are the bands at 1651, 1491, 1460 1423 and 1288 cm^−1^ coming from PVP and bands at 1150 and 1080 cm^−1^ coming from HPβCD. Finally, some bands characteristic of hesperidin/extract at 1032, 934 and 841 cm^−1^ are still visible with a slight wave shift.

#### 2.2.2. Characterization of Electrospun Nanofibers

Another important aspect in assessing electrospinning’s effect on nanofibers’ properties is the study of hesperidin content. The highest content was noted for the non-fiber system no. N1. A statistically significant relationship was shown that the increase in PVP concentration reduces the hesperidin content in the produced nanofibers (Table 5; Figure S9, Supplementary Materials).

**Figure 6:**
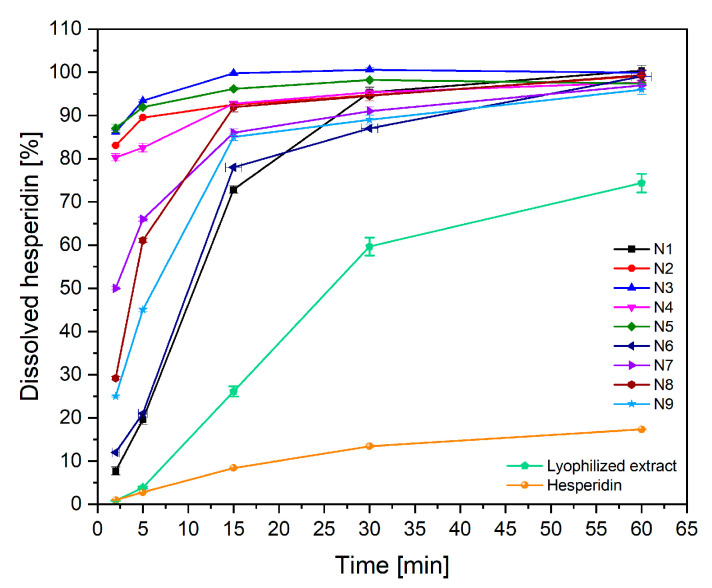
Dissolution profiles of hesperidin from nanofibers N1–N9 (n = 6).

Pure hesperidin is classified in the IV class according to the Biopharmaceutical Classification System, i.e., as a poorly soluble and poorly penetrating substance. The nanofibers are designed as a rapidly soluble form to enhance permeation within the oral cavity. Therefore, in order to increase the penetration of hesperidin through the oral epithelium directly into systemic circulation, it is essential that the compound dissolves quickly in oral liquids. Therefore, hesperidin release from the prepared nanofibers was assessed (Figure 6). The dissolution rate of pure hesperidin in 60 min was less than 20%, while at the same time, about 70% of hesperidin dissolved from the lyophilized extract. In the case of N2–N5 nanofibers, the release of hesperidin in 2 min was above 80%, which fulfilled the first goal of creating nanofibers as an innovative fast soluble form of hesperidin. There is no literature data regarding such a rapid dissolution of hesperidin from the delivery systems described so far. No statistically significant influence of the nanofiber preparation process on hesperidin release was observed (Figure S10, Supplementary Materials). Nevertheless, a correlation between the thickness of the nanofibers and the rate of hesperidin release was observed; a faster release of hesperidin from the thinnest nanofibers was noticed. This is obvious due to the larger wetting surface of thinner nanofibers [37].

**Figure 7:**
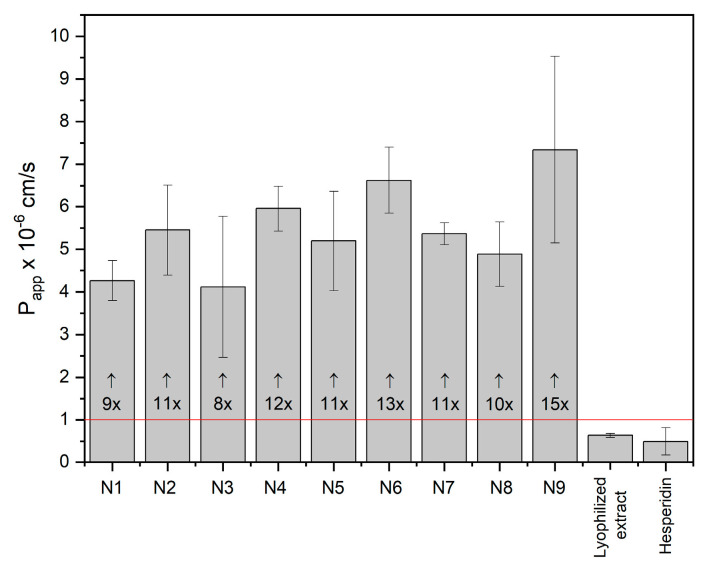
Permeability of hesperidin from nanofibers N1–N9 after 5 min (n = 6).

Scientists put considerable effort into improving the permeability of active compounds through biological membranes. One approach is to amorphize the compound. However, amorphization alone is not always sufficient to achieve this goal [16]. In the case of lyophilization of the extract, which also led to its amorphization, no significant improvement in the permeability of hesperidin through the membranes of the gastrointestinal tract was achieved. Importantly, in the case of the developed nanofibers, a more than 10-fold increase in the permeability of hesperidin through the membrane simulating the oral epithelium was achieved (Figure 7). It has been noted that a greater increase in permeability is observed in systems with higher HPβCD content (Figure S11, Supplementary Materials). HPβCD improves the solubility of active compounds, and those in the dissolved form are more available for transport across the membrane by passive diffusion. Consequently, one method to boost the penetration of bioactive compounds through biological membranes is to utilize cyclodextrins, which improve solubility and increase permeability through the undisturbed water layer [21,33].

Biological activity studies (antioxidant and anti-inflammatory activities tests) have also been performed for obtained nanofibers. The results obtained for the electrospun nanofibers remained at the same level as the results for the starting material, which show no change in the biological activity of the extract as an active ingredient. Therefore, the electrospinning process had a negative impact on the activity of the entire formulation.

**Figure 8:**
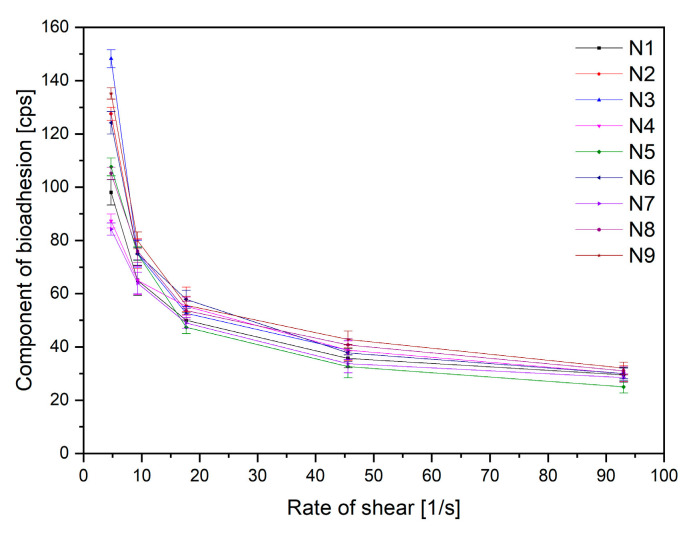
Mucoadhesive properties of nanofibers N1–N9 (n = 3).

Finally, an essential factor determining the application properties of the buccal preparation is the ability of the substance (PVP and HPβCD) to bind to the mucin found in the mucosa, which ensures that the product remains on the oral mucosa and does not cause the swallowing of the product into the digestive tract. For this purpose, rheological tests of nanofiber mixtures were used, the results of which are presented in Figure 8. Based on the Pareto chart, it was noticed that an increase in PVP concentration results in an increase in mucoadhesive properties. An inverse relationship was observed for HPβCD, where an increase in concentration causes a decrease in the viscosity of the systems (Figure S12, Supplementary Materials). The results are not surprising, as information about the mucoadhesive properties of PVP is reported in the literature [38]. The positive effect of cyclodextrins in this respect has not been demonstrated; however, other derivatives, i.e., thiolated cyclodextrins, have the potential to enhance the mucoadhesive by improving the viscosity of mucus [39].

**Figure 9:**
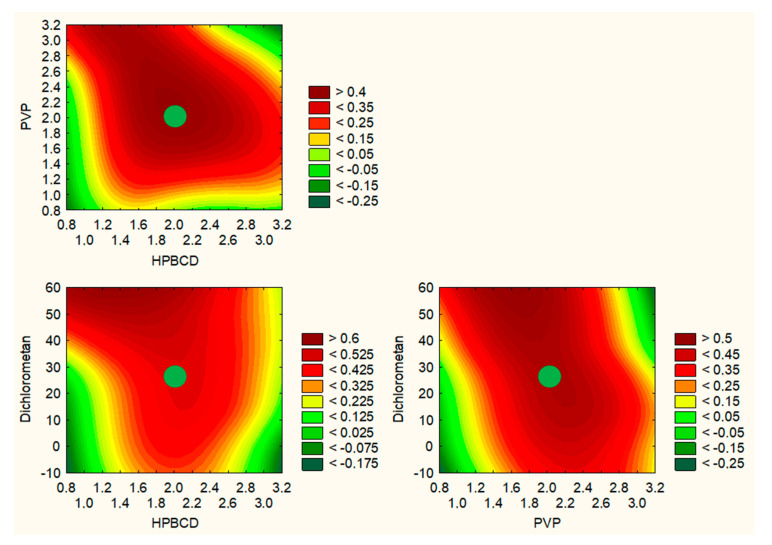
Prediction of DoE model of optimization of electrospinning process of nanofibers with orange peel extract.

To summarize the production step of electrospun nanofibers, a model of utility contours of profiles was made for all the tested effects (Figure 9). Based on the analysis of the following charts, the best properties were obtained for nanofibers with the composition of 2 g HPβCD/2 g PVP/25% dichloromethane/10 mL methanol, i.e., nanofiber no. 5. They are midway between the best chemical properties resulting from the high content of cyclodextrin and the best mechanical properties due to the high content of PVP.

## 3. Materials and Methods

### 3.1. Plant Raw Material

*Aurantii amari pericarpium et mesocarpium* (orange peel) was purchased from Natura Wita, Zdrowie z Zielnika (Pinczow, Poland) (lot no. 270821).

### 3.2. Chemicals and Reagents

Hesperidin (phyproof^®^ Reference Substance), hesperetin (≥95%), naringenin (98%), naringin (≥95%, HPLC), (+)-catechin (≥98%, HPLC), (−)-epicatechin (≥98%, HPLC), quercetin (≥95%, HPLC), rutin (≥94%, HPLC), kaempferol (≥97%, HPLC), caffeic acid (≥98%, HPLC) and luteolin (≥95%, HPLC) were obtained from Sigma-Aldrich (Poznan, Poland). Excipients, such as (2-Hydroxypropyl)-β-cyclodextrin (HPβCD), average Mw ~1460, were supplied from Sigma-Aldrich (Poznan, Poland), and polyvinylpyrrolidone (PVP) as Kollidon^®^ 30 was supplied from BASF Pharma (Burgbernheim, Germany). Reagents for activity assays include the following: 2,2-Diphenyl-1-picrylhydrazyl (DPPH), potassium persulfate (K_2_S_2_O_8_), 2,2′-Azino-bis(3-ethylbenzothiazoline-6-sulfonic acid) diammonium salt (ABTS, C_18_H_24_N_6_O_6_S_4_), neocuproine, ammonium acetate, copper(II) chloride (CuCl_2_·H_2_O), sodium acetate trihydrate (CH_3_COONa·3H_2_O), 2,4,6-tris(2-pyridyl)-1,3,5-triazine (TPTZ, C_18_H_12_N_6_), iron(III) chloride hexahydrate (FeCl_3_·6H_2_O), sodium chloride, bovine serum, hexadecyltrimethylammonium bromide (CTAB) and hyaluronic acid (HA); for dissolution studies, they include the following: potassium chloride, sodium chloride, di-potassium hydrogen orthophosphate, magnesium chloride, calcium chloride and xylitol; and for mucoadhesive tests, they include the following: mucin from porcine stomach obtained from Sigma-Aldrich (Poznan, Poland). Prisma™ HT buffer, Acceptor Sink Buffer and GIT lipid solution were obtained from Pion Inc. (Billerica, MA, USA), whereas HPLC-grade acetonitrile and water were obtained from Merck (Darmstadt, Germany). High-quality pure water and ultra-high-quality pure water were prepared using a Direct-Q 3 UV Merck Millipore purification system (Darmstadt, Germany).

### 3.3. Optimization of the Orange Peel Extraction Process and Characterization of Biological Activity of Extracts

#### 3.3.1. Plant Extraction Using Design of Experiment (DoE)

Using the Design of Experiments (DoE) approach, a factor experiment plan was developed for three independent variables, which were assigned three levels of values (3^2^ full factorial design). Independent factors were selected as the content of the extraction mixture, its temperature and the number of repetitions of the cycles (Table 6). A total of 5.0 g of plant material was extracted according to the experiment matrix using an ultrasonic bath, yielding a final extract concentration of 100 mg/mL.

The parameters used to assess extraction efficiency were chosen as hesperidin content, the total content of phenolic compounds and antioxidant (DPPH scavenging assay) as well as anti-inflammation activities (inhibition of hyaluronidase activity).

#### 3.3.2. Determination of Selected Active Component Content and Total Phenolic Content (TPC)

The contents of 11 active polyphenolic compounds (flavan-3-ols: catechin and epicatechin; flavanols: quercetin, rutin and kaempferol; phenolic acids: caffeic acid; flavanones: hesperetin, hesperidin, naringenin and naringin; and flavone: luteolin) were determined by using the modified HPLC-diode array detection method. As for equipment, the LC system (Dionex Thermoline Fisher Scientific) with Chromeleon software version 7.0 was used. Separations were performed on a LiChrospher RP-18 column, 5 μm particle size, 250 mm × 4 mm (Merck, Darmstadt, Germany). The mobile phase was composed of formic acid 0.1% (A) and acetonitrile (B) with a gradient elution: 0–10 min, 12% B; 10–60 min, 12–60% B; and 60–65 min, 12% B. The flow rate of the mobile phase was 0.6 mL/min, and the column temperature was maintained at 30 °C. The detection was performed using a diode array detector at wavelength maxima (*λ*;max) of 280, 330 and 360 nm.

The total content of phenolic components was determined by using a method described previously [40].

#### 3.3.3. Determination of Biological Activity

##### Antioxidant Activity

Antioxidant activity was determined by using an assay with 2,2-Diphenyl-1-picrylhydrazyl (DPPH), 2,2-Azino-bis(3-ethylbenzothiazoline-6-sulfonic Acid) (ABTS) radical cation-based assays, Cupric ion reducing antioxidant capacity (CUPRAC) assay and Ferric ion reducing antioxidant parameter (FRAP) assay. All procedures were described previously [40].

##### Anti-Hyaluronidase Activity

The procedure of hyaluronidase inhibition was determined by the turbidimetric method described previously [40].

### 3.4. Optimization of the Electrospinning Process of Nanofibers with Orange Peel Extract

#### Electrospun Nanofibers Preparation Using Design of Experiment (DoE)

The electrospinning procedure was performed using NS + NanoSpinner Plus Electrospinning Equipment (Inovenso Ltd., Istanbul, Turkey). The high voltage was set at 27 kV, the flow rate of the solution was 2 mL/h, and the distance between the syringe and rotary collector covered with aluminum foil was fixed at 12 cm. The experiments were carried out at room temperature (about 25 °C), and the humidity did not exceed 40%.

The amount of HPβCD and PVP and the solvent composition used to prepare nanofibers were selected based on Design of Experiment (DoE) data and the 3^2^ full factorial design experimental plan and are presented in Table 7. The parameters used to assess electrospinning efficiency were the diameter of nanofibers, hesperidin content, the total amount of the released active substance, hesperidin permeability and the bioadhesion properties of systems.

### 3.5. The Identification of Optimized Electrospun Nanofibers

#### 3.5.1. Dynamic Viscosity

The dynamic viscosity of solutions was measured with a Brookfield DV2T viscometer at a predetermined temperature (25 °C).

#### 3.5.2. Scanning Electron Microscopy (SEM)

The surface morphology of the nanofiber was visualized using SEM. The nanofibers were sputter-coated with gold–palladium and then visualized by a scanning electron microscope (Quanta 250 FEG, FE).

The diameter of nanofibers was measured on SEM images.

#### 3.5.3. XRPD

The crystallographic structure of the samples was analyzed by X-ray diffraction (XRD, Panalytical Empyrean, Almelo, Netherlands) equipment with the copper anode (CuKα—1.54 Å) at a Brag–Brentano reflection mode configuration with 45 kV and 40 mA parameters. The measurement parameters were set up for 3–60° with a 45 s. per a step size of 0.05° in all cases.

#### 3.5.4. Attenuated Total Reflectance Infrared Spectroscopy (ATR-IR)

The ATR-IR spectra were measured between 400 cm^−1^ and 4000 cm^−1^, with the resolution set to 1 cm^−1^, with a Shimadzu IRTracer-100 spectrometer equipped with a QATR-10 single bounce—diamond extended range and LabSolution IR software.

### 3.6. Characterization of Electrospun Nanofibers

#### 3.6.1. Determination of Active Component Content

Hesperidin content was determined by using the HPLC method described in Section 3.3.2.

#### 3.6.2. Dissolution Studies

Dissolution studies of electrospun nanofibers were performed using an Agilent 708-DS dissolution apparatus. A standard basket method was used at 37 ± 0.5 °C with a stirring speed of 50 rpm. Nanofibers were placed in 300 mL of artificial saliva solution at pH 6.8 (potassium chloride (1.20 g), sodium chloride (0.85 g), di-potassium hydrogen orthophosphate (0.35 g), magnesium chloride (0.05 g), calcium chloride (0.20 g), xylitol (20.0 g) and water up to 1 L; pH was adjusted to 6.8 by 1 M HCl). The liquid samples were collected at specified time intervals, and an equal volume of temperature-equilibrated media was replaced. The samples were filtered through a 0.45 μm nylon membrane filter. The HPLC method described above determined the concentrations of hesperidin in the filtered acceptor solutions. Sink conditions were preserved in the studies. The study was repeated six times.

#### 3.6.3. Permeability Studies

The permeability of active compounds enclosed in nanofibers through artificial biological membranes was investigated by using the gastrointestinal tract (GIT) PAMPA™ (parallel artificial membrane permeability assay) (Pion Inc., Billerica, MA, USA). Nanofibers were dissolved in donor solutions (artificial saliva solution at pH 6.8). The acceptor plates were loaded with an acceptor Prisma buffer at pH 7.4. The plates were put together and incubated at 37 °C for 15 min with continuous stirring at 50 rpm. Each experiment was repeated at least three times. The amount of permeated active compounds was determined using the HPLC method described above. The study was repeated six times.

The apparent permeability coefficients (*P_app_*) were calculated from the following equation:

$$P_{app}=\frac{-ln\left(1-\frac{C_{A}}{C_{equilibrium}}\right)}{S\times \left(\frac{1}{V_{D}}+\frac{1}{V_{A}}\right)\times t}$$

where *V_D_*—donor volume, *V_A_*—acceptor volume, *C_equilibrium_*—equilibrium concentration $C_{equilibrium}=\frac{C_{D}\times V_{D}+C_{A}\times V_{A}}{V_{D}+V_{A}}$, *C_D_*—donor concentration, *C_A_*—acceptor concentration, *S*—membrane area and *t*—incubation time (in seconds).

#### 3.6.4. Determination of Biological Activity

The biological activity of electrospun nanofibers was determined by assays described in Section 3.3.3.

#### 3.6.5. In Vitro Assessment of Mucin–Biopolymer Bioadhesive Bond Strength

A viscometric method was used to quantify mucin–polymer bioadhesive bond strength. The evaluation was performed according to the method described previously [41]. The study was repeated three times.

### 3.7. Statistical Analysis

Statistical analysis was carried out with Statistica 13.3 software. The normality of the results was checked using the Shapiro–Wilk test. The differences among the mean values were tested using the ANOVA test with post hoc Tukey’s range test for multiple comparisons. Differences between groups were considered to be significant at *p* < 0.05.

## 4. Conclusions

In the last few years, increased attention has been focused on industrial wastes, especially those containing residual phenols from used plant material. Therefore, the orange peel was selected for the study, which, in addition to being food waste, is also a good source of hesperidin. Based on the results of the DoE approach, it was possible to predict that the extraction efficiency increases with the increase in the percentage of methanol in the extraction mixture and temperature. Optimized parameters of the orange peel extraction process were selected as 70% of methanol in the extraction mixture, a temperature of 70 °C and 4 cycles per 20 min.

Moreover, this study found that hesperidin contained in orange peel extract can be complexed with HPβCD/PVP and subjected to the electrospinning procedure to form stable nanofibers. The HPβCD/PVP-based formulation as well as the electrospinning process affected the solubility of hesperidin nanofibers, which significantly increased. In addition, an over nine-fold increase in the permeability of hesperidin contained in nanofibers was demonstrated. The best properties were obtained for nanofibers with the composition of HPβCD/PVP/25% dichloromethane/10 mL methanol, i.e., nanofiber no. N5. Therefore, hesperidin-loaded nanofibers can effectively improve hesperidin’s aqueous solubility and show enhanced penetration through the biological membrane, with the maintenance of its antioxidant and anti-inflammatory properties. Therefore, hesperidin-rich orange peel nanofibers may have potential buccal applications to induce improved systemic effects with pro-health biological activity.

## Figures and Tables

**Table 1:** Content of active compounds in extracts E1–E9.

	Content of Active Compounds	
No.	Content of Hesperidin (mg/g Plant Material)	TPC (mg GAE/g Plant Material)
E1	10.17 ± 0.92	5.34 ± 1.33
E2	10.33 ± 0.52	5.85 ± 1.34
E3	27.80 ± 0.13	5.89 ± 1.31
E4	12.14 ± 0.75	6.64 ± 0.62
E5	24.40 ± 0.09	6.77 ± 0.51
E6	27.74 ± 0.01	6.73 ± 0.75
E7	23.15 ± 0.96	9.58 ± 2.25
E8	28.45 ± 0.54	9.92 ± 2.79
E9	33.73 ± 0.54	11.01 ± 1.17

**Table 2:** Antioxidant and anti-inflammatory activity of extracts E1–E9.

	Antioxidant Activity				Anti-Inflammatory Activity
No.	DPPH IC_50_ (mg/mL)	ABTS IC_50_ (µg/mL)	CUPRAC IC_0.5_ (µg/mL)	FRAP IC_0.5_ (µg/mL)	IC_0.5_ (µg/mL)
E1	2.82 ± 0.07	760.90 ± 23.65	1670.37 ± 34.84	500.18 ± 48.94	21.87 ± 6.28
E2	2.41 ± 0.20	632.99 ± 8.56	1673.53 ± 13.09	498.09 ± 79.76	32.38 ± 4.45
E3	2.18 ± 0.34	567.31 ± 16.76	1560.13 ± 74.88	487.65 ± 82.75	43.41 ± 4.11
E4	2.35 ± 0.36	546.32 ± 44.24	696.91 ± 109.96	482.12 ± 9.80	54.78 ± 12.07
E5	2.22 ± 0.00	535.17 ± 30.63	797.48 ± 117.39	469.29 ± 38.55	81.12 ± 1.56
E6	2.11 ± 0.04	479.29 ± 14.77	860.33 ± 76.27	456.33 ± 61.61	84.80 ± 0.00
E7	2.27 ± 0.07	603.92 ± 45.75	682.87 ± 57.92	485.52 ± 18.56	>1000
E8	2.13 ± 0.04	471.58 ± 13.84	764.48 ± 104.93	449.87 ± 54.57	>1000
E9	1.93 ± 0.22	338.32 ± 5.36	705.62 ± 145.90	436.02 ± 13.51	>1000

**Table 3:** Diameter of nanofibers N1–N9.

N1	N2	N3	N4	N5	N6	N7	N8	N9
Diameter of nanofibers (µm)								
-	0.67 ± 0.19	1.14 ± 0.55	0.75 ± 0.33	0.94 ± 0.30	1.33 ± 0.39	4.53 ± 3.74	2.73 ± 1.12	1.18 ± 0.46

**Table 4:** The efficiency of creating nanofibers.

N1	N2	N3	N4	N5	N6	N7	N8	N9
%								
20.79	53.71	41.49	42.25	50.52	1.12	1.17	4.40	0.54

**Table 5:** Content of hesperidin in nanofibers (n = 3).

N1	N2	N3	N4	N5	N6	N7	N8	N9
Content µg in 100 mg of nanofibers								
522.90 ± 13.19	182.86 ± 0.28	115.91 ± 1.59	199.50 ± 0.44	233.46 ± 0.80	158.70 ± 1.88	212.80 ± 0.43	164.03 ± 0.46	65.74 ± 0.03

**Table 6:** Factorial Extraction Process Experiment Plan for extraction process.

No.	% of Methanol in the Extraction Mixture	Temperature	Number of Cycles
E1	0	30	3
E2	0	50	5
E3	0	70	4
E4	35	30	5
E5	35	50	4
E6	35	70	3
E7	70	30	4
E8	70	50	3
E9	70	70	5

**Table 7:** Factorial Extraction Process Experiment Plan for electorspinning process.

No.	HPBCD (g)	PVP (g)	Solvent Composition (Percentage of Methanol; Supplemented with Dichloromethane)
N1	1	1	100
N2	1	2	50
N3	1	3	75
N4	2	1	50
N5	2	2	75
N6	2	3	100
N7	3	1	75
N8	3	2	100
N9	3	3	50

## Data Availability

The data are contained within the article and Supplementary Materials.

## Supplementary Materials

The following supporting information can be downloaded at: https://www.mdpi.com/article/10.3390/ijms24097963/s1.
