# Supplementary material for: Improving Solubility and Permeability of Hesperidin through Electrospun Orange-Peel-Extract-Loaded Nanofibers
Source: Int J Mol Sci. 2023 Apr 27;24(9):7963. doi: 10.3390/ijms24097963 (PMC10178203; doi:10.3390/ijms24097963)
Supplement: Supplementary file 1 [file ijms-24-07963-s001.zip › ijms-2318257-supplementary.pdf]

Electrospun nanofibers containing orange peel extract as an innovative system that improves the solubility and permeability of hesperidin

Supplementary material

Table S1. HPLC method validation parameters

	flavan-3-ols		flavanols			phenolic acids	flavanones				flavone
	catechin	epicatechin	quercetin	rutin	kaempferol	caffeic acid	hesperetin	hesperidin	naringenin	naringin	luteolin
Detection wavelength [nm]	280	280	360	360	360	330	280	280	280	280	330
Linerity<br>$a \pm S_a$<br>$b \pm S_b$	0.174 ± 0.001<br>insignificant	0.108 ± 0.001<br>insignificant	0.521 ± 0.025<br>insignificant	0.225 ± 0.002<br>insignificant	0.463 ± 0.006<br>insignificant	0.809 ± 0.024<br>insignificant	0.442 ± 0.011<br>insignificant	0.102 ± 0.006<br>insignificant	0.400 ± 0.018<br>insignificant	0.242 ± 0.004<br>insignificant	0.512 ± 0.022<br>insignificant
Correlation coefficient (r)	0.9990	0.9998	0.9981	0.9999	0.9998	0.9986	0.9991	0.9864	0.9966	0.9996	0.9971
Linearity range [µg/ml]	120 - 600	162 - 810	113 - 452	251 - 1255	100 - 500	146 - 730	174 - 870	830 - 4150	345 - 1725	200 - 1000	125 - 625
Limit of Detection (LOD) [µg/mL]	34.029	19.940	39.887	24.886	12.875	46.144	45.246	488.753	161.755	36.449	57.006
Limit of quantification (LOQ) [µg/mL]	103.117	60.424	120.868	75.412	39.014	139.831	137.108	1481.069	490.166	110.452	172.745

S_a - standard deviation of the slope; S_b - standard deviation of the intersection point. t. calculated values of the Student's t-test. $t\alpha.f$ = 3.182 Critical values of the Student's test for degrees of freedom f = 3 and significance level α = 0.05.

Table S2. The content of active compounds

	flavan-3-ols			flavanols		phenolic acids		flavanones			flavone
	catechin	epicatechin	quercetin	rutin	kaempferol	caffeic acid	hesperetin	hesperidin	naringenin	naringin	luteolin
E1	n/d	1335.95 ± 20.48	79.07 ± 32.07	28.55 ± 7.94	8.14 ± 4.90	129.69 ± 8.13	348.48 ± 11.68	10.17 ± 0.92	384.64 ± 12.92	n/d	116.04 ± 49.68
E2	n/d	3587.01 ± 18.37	224.31 ± 13.46	2703.39 ± 674.78	13.79 ± 1.70	353.20 ± 2.22	129.70 ± 16.88	10.33 ± 0.52	219.39 ± 13.76	n/d	410.64 ± 5.95
E3	n/d	1144.80 ± 183.28	71.33 ± 6.02	575.82 ± 345.59	1.93 ± 0.30	92.82 ± 52.23	20.24 ± 3.62	27.80 ± 0.13	4.47 ± 1.55	n/d	125.42 ± 11.33
E4	n/d	3961.91 ± 82.39	263.09 ± 22.26	2449.25 ± 656.65	29.94 ± 3.02	436.80 ± 80.36	174.84 ± 25.07	12.14 ± 0.75	41.27 ± 15.00	n/d	505.09 ± 80.54
E5	n/d	2961.28 ± 1475.93	176.56 ± 64.78	1711.42 ± 631.21	11.70 ± 8.07	296.58 ± 106.33	159.06 ± 14.06	24.40 ± 0.09	329.32 ± 462.02	n/d	305.07 ± 124.84
E6	n/d	3485.18 ± 251.16	219.18 ± 2.69	2409.94 ± 209.47	5.30 ± 0.24	393.80 ± 45.70	24.45 ± 5.85	27.74 ± 0.01	42.89 ± 0.66	n/d	374.85 ± 7.18
E7	n/d	2690.16 ± 5.50	197.58 ± 3.22	1914.89 ± 14.93	14.05 ± 2.52	321.63 ± 20.45	18.34 ± 1.52	23.15 ± 0.96	238.24 ± 4.05	n/d	346.93 ± 5.31
E8	n/d	1298.95 ± 110.82	63.79 ± 23.62	825.97 ± 378.86	5.00 ± 1.80	141.44 ± 9.34	131.80 ± 21.75	28.45 ± 0.54	3.61 ± 1.45	n/d	110.04 ± 41.31
E9	n/d	3238.25 ± 637.20	138.81 ± 81.45	1294.15 ± 1109.40	7.37 ± 1.27	318.12 ± 25.44	6.14 ± 2.39	33.73 ± 0.54	2.92 ± 3.44	n/d	324.90 ± 36.31

n/d - non-detected

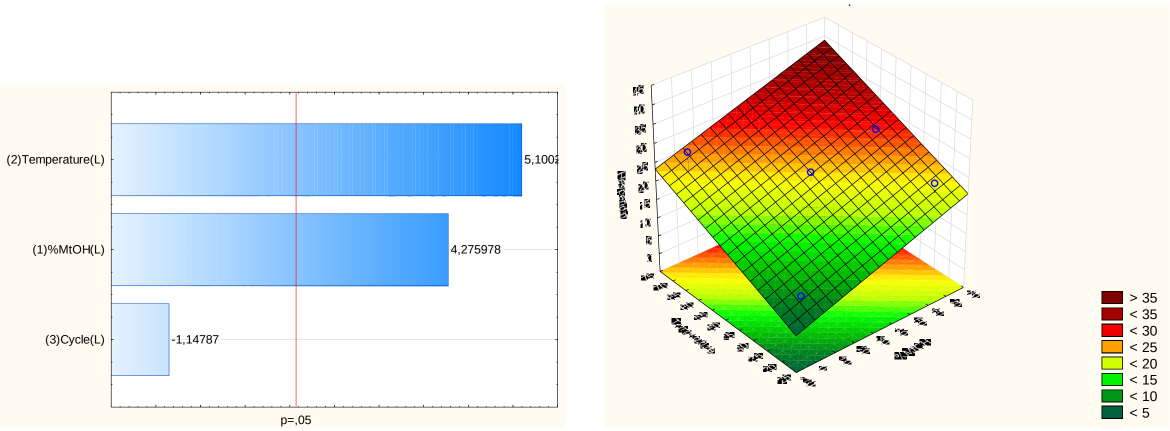

(a) (b)

Figure S1. Statistical analysis for hesperidin content in extracts E1-E9: (a) Pareto plot of standardized effects for hesperidin content in extracts E1-E9; (b) Response surface plots presenting the dependence of methanol content in the extraction mixture and extraction temperature on the hesperidin content in extracts for constant number of extraction cycles at level 4.

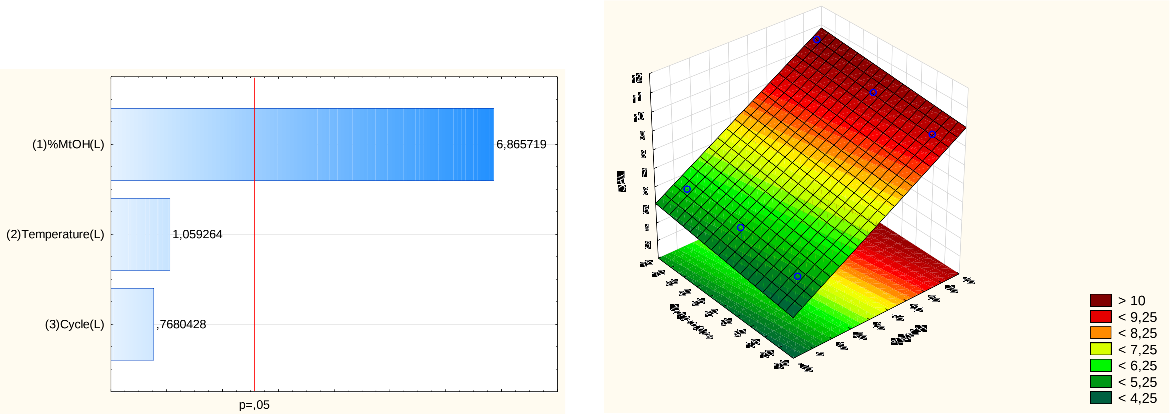

(a) (b)

Figure S2. Statistical analysis for total phenolic content in extracts E1-E9: (a) Pareto plot of standardized effects for total phenolic content in extracts E1-E9; (b) Response surface plots presenting the dependence of methanol content in the extraction mixture and extraction temperature on the total phenolic content in extracts for constant number of extraction cycles at level 4.

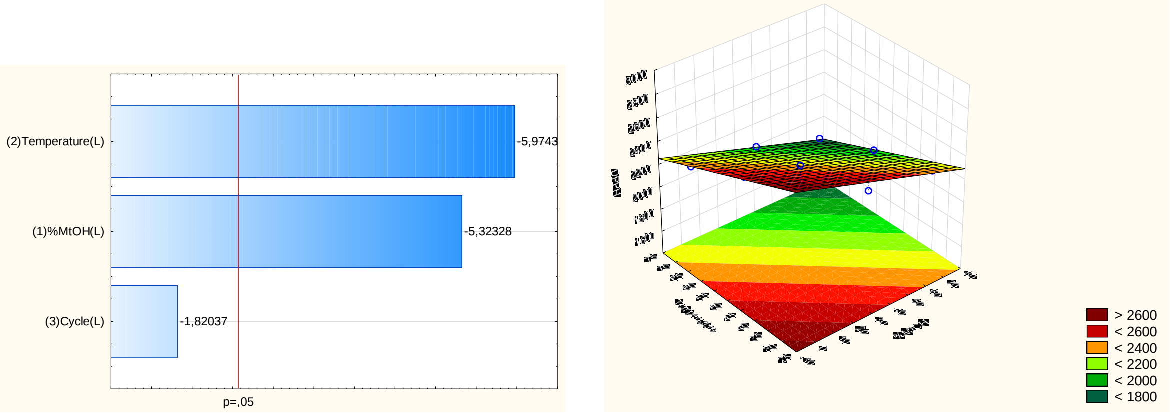

(a) (b)

Figure S3. Statistical analysis for antioxidant activity of extracts E1-E9 measured by DPPH method: (a) Pareto plot of standardized effects for antioxidant activity; (b) Response surface plots presenting the dependence of methanol content in the extraction mixture and extraction temperature on antioxidant activity of extracts for constant number of extraction cycles at level 4.

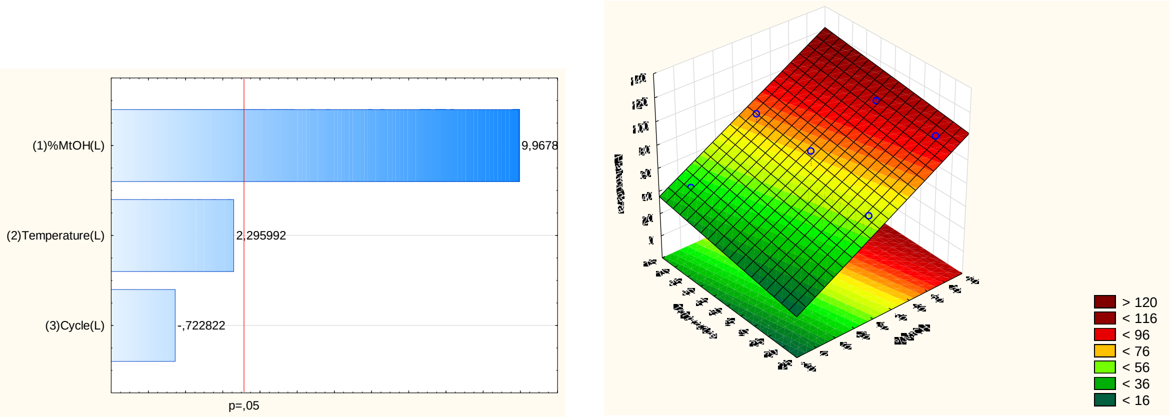

(a) (b)

Figure S4. Statistical analysis for anti-inflammatory activity of extracts E1-E9: (a) Pareto plot of standardized effects for anti-inflammatory activity of extracts E1-E9; (b) Response surface plots presenting the dependence of methanol content in the extraction mixture and extraction temperature on anti-inflammatory activity of extracts for constant number of extraction cycles at level 4.

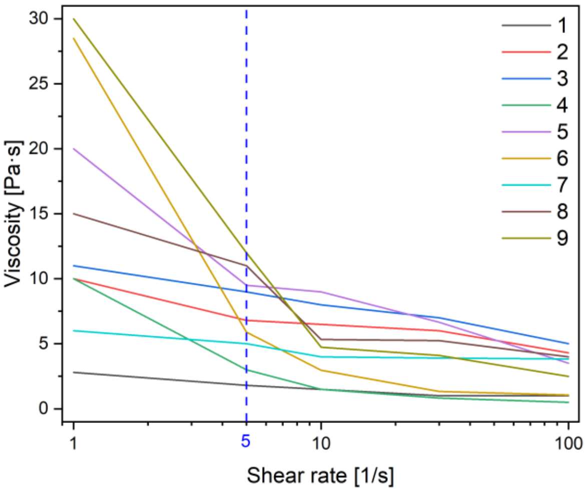

Figure S5. Rheological behavior of the prepared solutions for electrospinning – relationships between viscosities and shear rates.

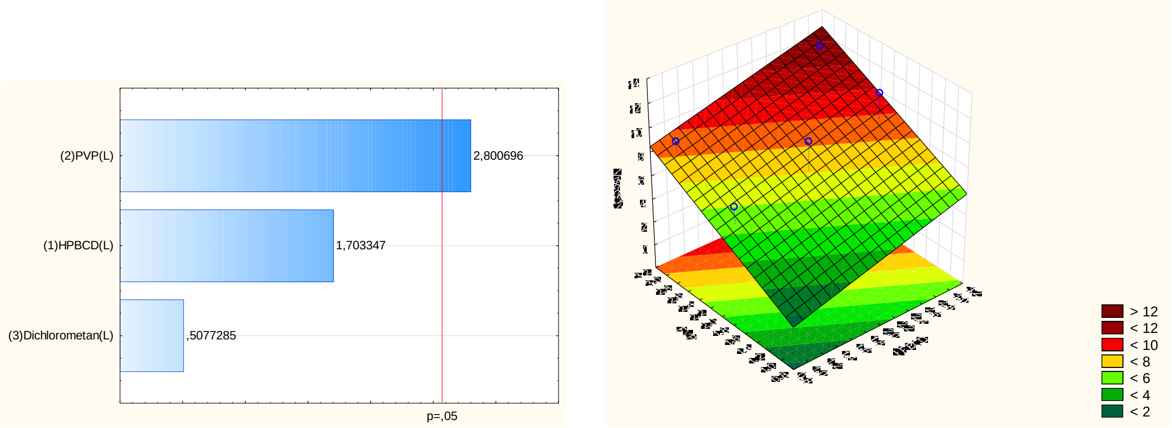

(a) (b)

Figure S6. Statistical analysis for viscosities of the prepared solutions for electrospinning: (a) Pareto plot of standardized effects for viscosities of the prepared solutions for electrospinning; (b) Response surface plots presenting the dependence of HPβCD and PVP concentration on the viscosities of the prepared solutions for electrospinning for constant concentration of dichloromethane at level 25%.

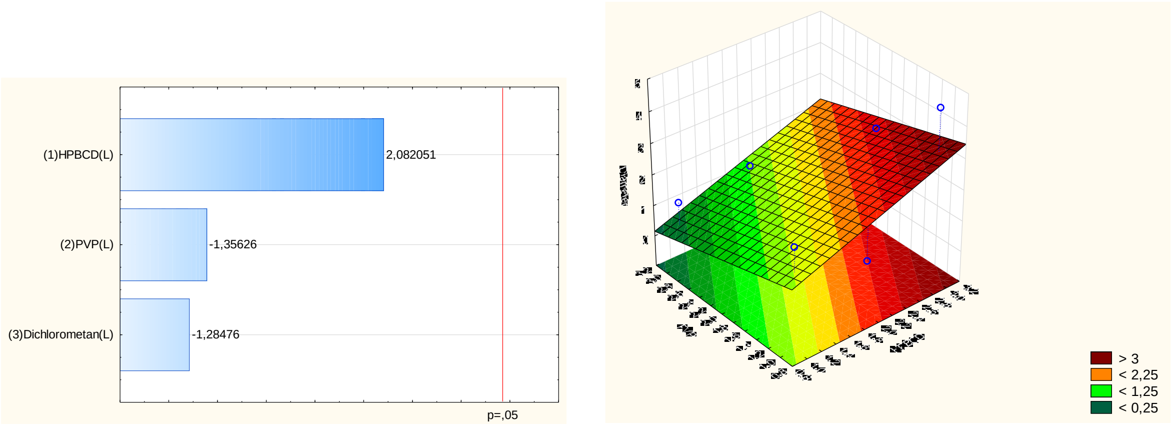

(a) (b)

Figure S7. Statistical analysis for diameter of nanofibers: (a) Pareto plot of standardized effects for diameter of nanofibers N1-N9; (b) Response surface plots presenting the dependence of HPβCD and PVP concentration on the diameter of nanofibers for constant concentration of dichloromethane at level 25%.

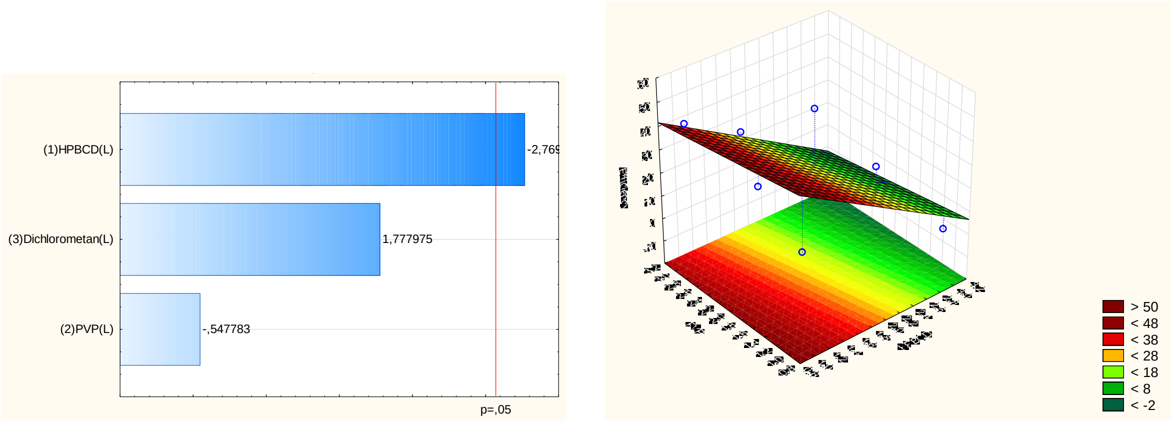

(a) (b)

Figure S8. Statistical analysis for efficiency of nanofiber production: (a) Pareto plot of standardized effects for efficiency of nanofiber production N1-N9; (b) Response surface plots presenting the dependence of HPβCD and PVP concentration on the efficiency of nanofiber production for constant concentration of dichloromethane at level 25%.

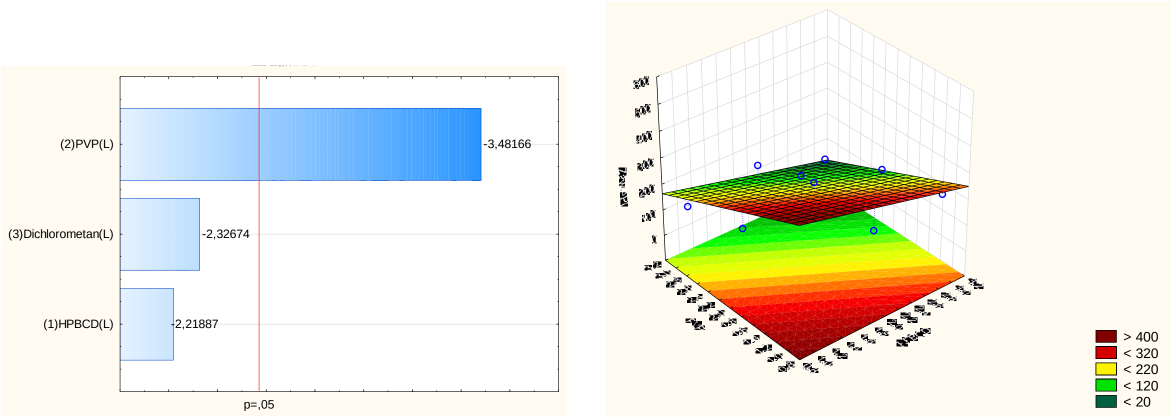

(a) (b)

Figure S9. Statistical analysis for hesperidin content: (a) Pareto plot of standardized effects for hesperidin content in nanofibers N1-N9; (b) Response surface plots presenting the dependence of HPβCD and PVP concentration on the hesperidin content for constant concentration of dichloromethane at level 25%.

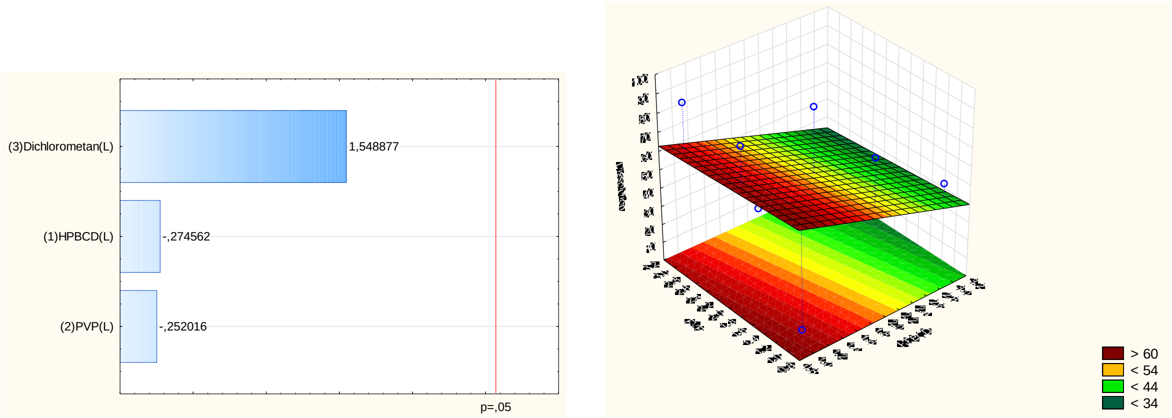

(a) (b)

Figure S10. Statistical analysis for hesperidin dissolution: (a) Pareto plot of standardized effects for hesperidin dissolution in 2 minutes from nanofibers N1-N9; (b) Response surface plots presenting the dependence of HPβCD and PVP concentration on the hesperidin dissolution for constant concentration of dichloromethane at level 25%.

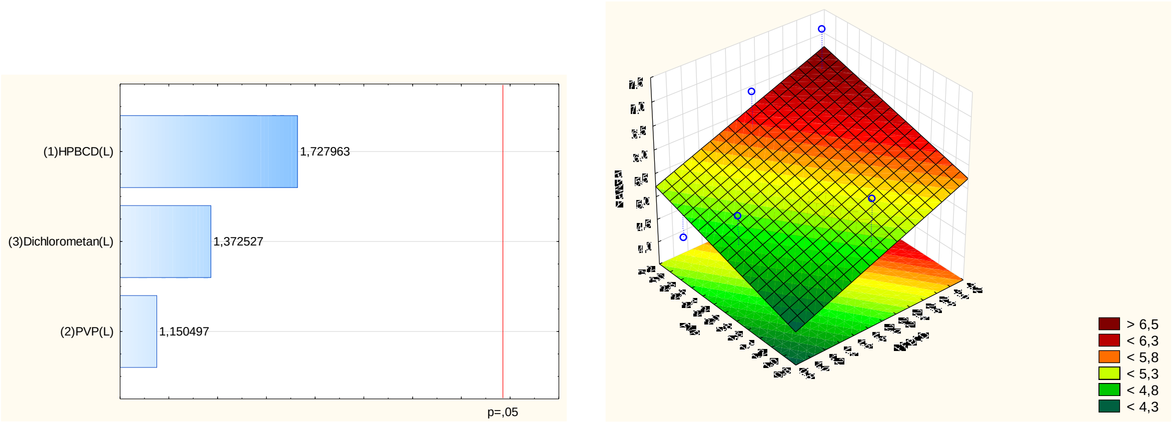

(a) (b)

Figure S11. Statistical analysis for hesperidin permeability: (a) Pareto plot of standardized effects for hesperidin permeability from nanofibers N1-N9; (b) Response surface plots presenting the dependence of HPβCD and PVP concentration on the hesperidin permeability for constant concentration of dichloromethane at level 25%.

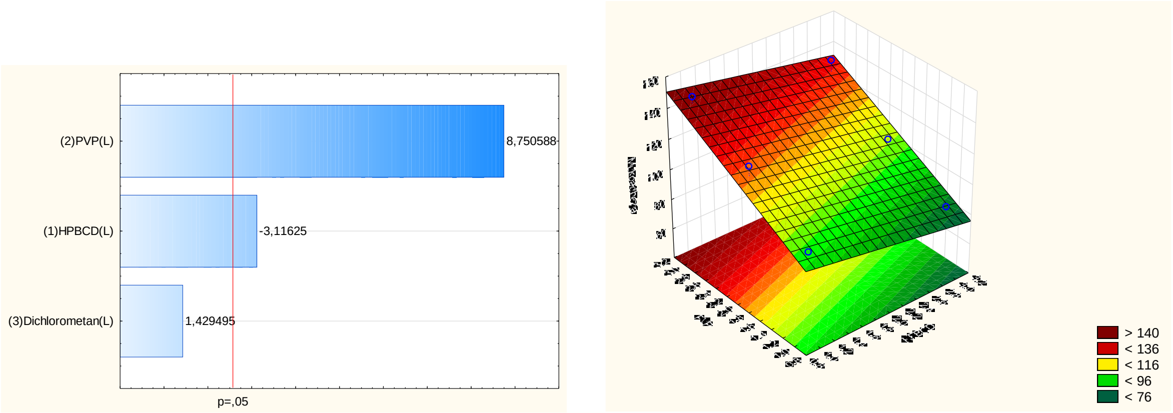

(a) (b)

Figure S12. Statistical analysis for component of mucoadhesion: (a) Pareto plot of standardized effects for mucoadhesive properties of nanofibers N1-N9; (b) Response surface plots presenting the dependence of HPβCD and PVP concentration on the component of mucoadhesion for constant concentration of dichloromethane at level 25%.
